# 流式细胞术检测CD45表达缺失的结外鼻型NK/T细胞淋巴瘤1例

**DOI:** 10.3760/cma.j.cn121090-20231130-00285-1

**Published:** 2024-08

**Authors:** 晨曦 胡, 佳佳 代, 旭华 张, 敏 王, 小姣 汪, 旭东 魏

**Affiliations:** 郑州大学附属肿瘤医院（河南省肿瘤医院），郑州 450003 Affiliated Cancer Hospital of Zhengzhou University/Henan Cancer Hospital, Zhengzhou 450003, China

患者，男，31岁。2022年4月因鼻塞伴血性鼻涕于当地医院就诊，入院完善相关检查后，行鼻内窥镜下多窦口开放术、双中下鼻甲部分切除术、鼻窦肿物切除术与鼻中隔矫正术，术后病理结果示非霍奇金淋巴瘤，倾向NK/T细胞淋巴瘤。原位杂交EBER（+）。MRI：双侧鼻腔肿物切除术后改变，符合NK/T细胞淋巴瘤双侧鼻甲浸润。综上明确诊断为结外鼻型NK/T细胞淋巴瘤（ENKTL），2022年4月至7月于当地医院行P-GemOx（培门冬酶+吉西他滨+奥沙利铂）方案2个周期、放疗25次，11月复查提示病情稳定，12月出现发热，淋巴结进行性增大，病理活检及PET-CT结果显示复发并累及淋巴结，患者为进一步治疗就诊于我院。全身CT及PET-CT示鼻中隔前部、双侧下鼻甲黏膜增厚；颈部双侧小淋巴结、左侧腹股沟及左侧腘窝多发软组织结节及肿块影；双肺多发磨玻璃密度结节。胃体壁多处不均匀，代谢增高，全身多处骨骼代谢增高，考虑淋巴瘤浸润，疾病进展。

2023年3月患者化疗（阿扎胞苷+门冬酰胺酶+地塞米松+米托蒽醌脂质体+PD-1单抗）后复查，骨髓细胞学检查提示幼淋样细胞占12.00％，流式细胞术（FCM）免疫分型结果显示：CD45^+^异常NK淋巴细胞群占有核细胞的19.90％，表达CD45、CD7、CD2，强表达CD56，弱表达CD26、CD45RO，不表达CD5、CD3、CD4、CD8、CD25、CD30、CD57、CD16、CD45RA、TCRαβ、TCRγδ、CD10；CD45^−^异常NK淋巴细胞群占有核细胞的0.10％，表达CD7、CD2，强表达CD56，弱表达CD26，不表达CD45、CD5、CD3、CD4、CD8、CD25、CD30、CD57、CD16、CD45RA、CD45RO、TCRαβ、TCRγδ、CD10，符合ENKTL表型。建议治疗达到完全缓解后行allo-HSCT，患者及家属拒绝治疗并出院。4月下旬再次入我院进行化疗（阿扎胞苷+门冬酰胺酶+地塞米松+米托蒽醌脂质体+替雷利珠单抗），效果不佳，面部肿胀、眼睑水肿等临床症状无缓解。骨髓细胞学复查结果显示幼淋样细胞占3.20％，FCM结果：CD45^+^异常NK淋巴细胞群占有核细胞的0.40％，CD45^−^异常NK细胞群占有核细胞的2.24％，免疫表型较前一致。进一步检测异常细胞群，结果显示CD45^+^与CD45^−^异常NK细胞群均表达CD94、CD161、cTIA-1，其Ki-67表达率分别为56.06％和80.36％，不表达cGranzyme、cPerforin；NK-KIR检测示CD45^+^与CD45^−^异常NK细胞群CD158表达缺失，考虑为单克隆性增殖。5月底患者入GFH009单药临床试验组，用药1周后评估治疗无效后退出试验组，拒绝后续治疗出院，回当地未行特殊治疗后死亡。

讨论：本文报道的ENKTL病例，除鼻塞、血性鼻涕及EB病毒阳性等典型临床表现外，FCM结果显示该患者存在CD45^+^与CD45^−^两群异常NK细胞。有数据显示，ENKTL患者异常细胞多数表达CD45，约占83％，偶见CD45缺失。然而CD45^+^与CD45^−^异常NK细胞同时出现的病例十分罕见，本例两群异常NK细胞群随着治疗过程发生了优势克隆的转化。患者首次FCM结果显示异常NK细胞群以CD45^+^为主，弱表达CD45RO，提示该细胞群代谢增殖活跃，CD45^−^异常细胞群仅占有核细胞的0.10％，极易被忽略。然而随着治疗，该群CD45^+^优势克隆细胞比例逐渐降低，CD45^−^异常细胞群占比逐渐增高，细胞数量也逐渐升高，该细胞群逐渐由劣势克隆转变为优势克隆。此外，该群CD45^−^异常细胞Ki-67表达率相较CD45^+^细胞群明显增高（*χ*^2^ = 13.24，*P*<0.05），表明该群CD45^−^异常NK细胞恶性程度更高，可能是后续患者治疗效果不佳的原因。回顾该患者治疗过程及FCM检测结果，CD45^+^异常NK细胞群随着治疗比例减低，由优势克隆转化为劣势，可能与针对患者的治疗对CD45^+^异常细胞群效果较好，而对CD45^−^异常细胞群疗效不佳有关，该患者预后不好与该群CD45^−^细胞密切相关，CD45有作为ENKTL治疗效果及预后评估指标的潜力，也提醒在ENKTL的FCM诊断过程中应兼顾CD45^−^细胞群，以免误诊漏诊。

